# Systematic review of pancreatic cancer epidemiology in Asia-Pacific Region: major patterns in GLOBACON 2012 

**Published:** 2017

**Authors:** Mohamad Amin Pourhoseingholi, Sara Ashtari, Nastaran Hajizadeh, Zeinab Fazeli, Mohammad Reza Zali

**Affiliations:** 1 *Gastroenterology and Liver Diseases Research Center, Research Institute for Gastroenterology and Liver Diseases, Shahid Beheshti University of Medical Sciences, Tehran, Iran*; 2 *Basic and Molecular Epidemiology of Gastrointestinal Disorders Research Center, Research Institute for Gastroenterology and Liver Diseases, Shahid Beheshti University of Medical Sciences, Tehran, Iran*

**Keywords:** Pancreatic cancer, Epidemiology, Risk factors, Asia, Pacific

## Abstract

Pancreatic cancer is one of the deadliest cancers with short-term survival rates. Trends for pancreatic cancer incidence and mortality varied considerably in the world. To date, the causes of pancreatic cancer are not known sufficiently, although certain risk factors have been identified such as, smoking, obesity, life style, diabetes mellitus, alcohol, dietary factors and chronic pancreatitis. Since there are no current screening recommendations for pancreatic cancer, primary prevention is very important. Therefore, up-to-date statistics on pancreatic cancer occurrence and outcome are essential for the primary prevention of this disease. Due to the lack of information on epidemiology of pancreatic cancer in most Asian countries, and limited of statistics and registration system in this area, we conducted a systematic review study to evaluate the most recent data concerning epidemiology of pancreatic cancer in Asia-Pacific region. In this review we focused on collected recent data on incidence, mortality, survival and risk factors of pancreatic cancer in this region. In addition, we reviewed and used the data of GLOBOCAN 2012 in this paper to complete the information as a source of compiling pancreatic cancer incidence and mortality rate.

## Introduction

 Pancreatic cancer is a highly aggressive cancer and has ranked the 12th most common cancer in the world with 338,000 new cases and the 7th most frequent cause of cancer death worldwide with 331,000 deaths per year in both sexes in 2012 (1). The numbers of incidence cases and pancreatic cancer deaths are similar because prevention or early diagnosis at a curable stage is extremely difficult (2). Patients rarely exhibit symptoms in the early stages, so the disease is generally advanced when it is diagnosed (3). GLOBOCAN estimated 5-year prevalence of people in the world living with pancreatic cancer is 4.1 per 100,000 in 2012 (1). 

**Figure 1:**
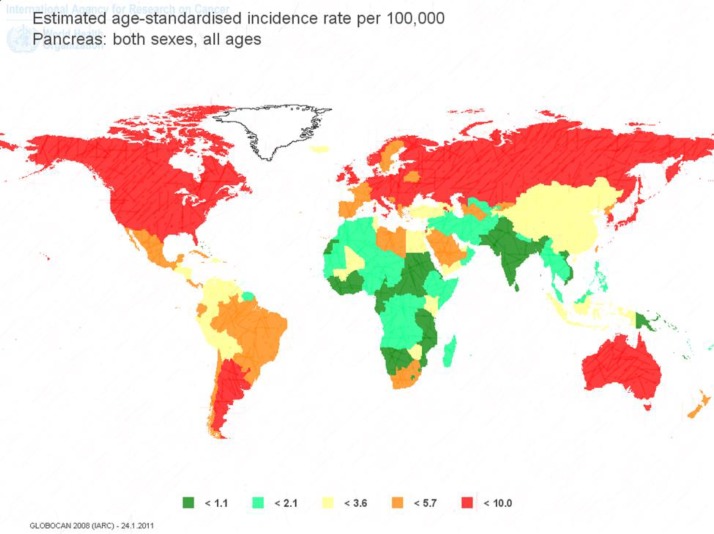
The age-standard incidence of pancreatic cancer in the world, based on GLOBOCAN 2012.

 The survival rate of pancreatic cancer is exceedingly low and the case fatality rate for the disease is approximately 0.97 that has declined slightly (from 0.99 to 0.97) last 12 years (4). In different areas of the world, pancreatic cancer is quiet infrequent, based on the GLOBOCAN 2012, the age-standardized rate (ASR) incidence is highest in North America (7.4/100,000) and then followed by Europe (6-7.3/100,000), Oceania (>6/100,000) and lowest in South-Central Asia and most of Africa (1.0/100,000) (5) as represented in figure 1.

The burden of the pancreatic cancer is increasing due to aging, population growth and increasing risk factors such as smoking, obesity, lifestyle, diabetes mellitus, alcohol and chronic pancreatitis (6, 7). Based on estimation of International Agency for Research on Cancer (IARC) on 2015, global pancreatic cancer rates could increase by 24% to 418,000 by 2020 (8).

Since there are no current screening recommendations for pancreatic cancer, primary prevention is very important. Better understanding of the epidemiology and identifying the risk factors of pancreatic cancer are essential for the primary prevention of this disease. Therefore, we conducted a systematic review study to evaluate the most recent data concerning on epidemiology of pancreatic cancer in Asia-Pacific region. The purpose of this review is to examine and gather recent data on pancreatic cancer with focus on incidence, mortality, survival and risk factors related to this disease in this region. Moreover, we used the data of GLOBOCAN 2012 in order to complete information, compare and finalize conclusion as a source of compiling pancreatic cancer incidence and mortality rate.

## Methods

We performed a comprehensive review according to the PRISMA guidelines. We chose articles published previously in Medline, PubMed, Scopus, Google Scholar, ISI Web of Knowledge (Science Citation Index Expanded) and Cochrane database. We searched previous studies on the epidemiology of pancreatic cancer, published up to 31 March 2017, with English language restriction. 

Search with the following MeSH terms and keywords included: “epidemiology of pancreatic cancer”, “incidence and mortality of pancreatic cancer” and “risk factors of pancreatic cancer” with specific risk factors such as: tobacco, alcohol, height, weight, diet, genetic, etc. Each study was cross-referenced with “Asia-Pacific region” and countries in this region such as Australia, New Zealand, India, Pakistan, Iran and others. We checked titles and abstracts and retrieved pertinent information from the full text of relevant studies. We also reviewed additional articles listed in the bibliography of retrieved publications. 

We selected those papers that considered being most important and appropriate. All relevant articles were accessed in full text and all relevant materials were evaluated and reviewed. Moreover, we used the GLOBOCAN 2012 data that published about pancreatic cancer in order to complete information, compare and finalize conclusion. All findings were reviewed and analyzed, then reported as results in the tables and text.

## Results

**Asia-Pacific region**

Asia is the most populous continent in the world and it covers approximately 4.3 billion people, about 60% of the world’s current population (9). Two most populated countries in the world were located in this continent; China with 1.4 billion people and India with 1.2 billion people (10). Even with a relatively low population growth rate of 0.9%, the Asia-Pacific region added more than 40 million people to its population during 2013-2014 (10).

Asia-Pacific region varies in size depending on context, but it typically includes East Asia, South Asia, Southeast Asia, and Oceania. To cover the entire Asia in this study, West Asia and Central Asia were also examined in this paper. 

**Incidence and Mortality of pancreatic cancer**

The incidence and mortality of pancreatic cancer varies greatly across regions and populations. GLOBOCAN in 2012 for Asia and Oceania estimated the age-standardized rate (ASR) incidence 3.2 and 5.9/100,000 and ASR mortality was 3.0 and 5.2/100,000 respectively (table 1) (5). Based on table 1, in 2012 Eastern and Western Asia (4.5 and 3.9/100,000 ASR) have higher incidence of pancreatic cancers compared to southeast and center of Asia (2.2 and 1.2/100,000 ASR). And the highest rate (6.5/100,000) of ASR incidence is in Australia and New Zealand. 

**Table 1:** The overall age-standardized incidence, mortality and 5-year prevalence rates from pancreatic cancer per 100,000 in different Asia regions and Oceania, based on GLOBACON 2012 (8)

Pancreatic Cancer		Incidence			Mortality			5-Yaer Prevalence		
Region		Number	(%)	ASR(W)*	Number	(%)	ASR(W)	Number	(%)	ASR(W)
Southeast Asia	Male Female Total	6413 5869 12282	1.7 1.5 1.6	2.5 2.0 2.2	6207 5651 11858	2.1 2.4 2.2	2.5 1.9 2.2	4866 4451 9317	0.8 0.4 0.6	2.2 2.0 2.1
Central Asia	Male Female Total	9299 7511 16810	1.3 0.9 1.1	1.3 1.0 1.2	8560 6999 15559	1.6 1.4 1.5	1.2 1.0 1.1	5661 4522 10183	0.5 0.3 0.4	0.9 0.7 0.8
Western Asia	Male Female Total	4013 2887 6900	2.4 1.9 2.2	4.7 3.1 3.9	3894 2823 6717	3.5 3.6 3.5	4.7 3.0 3.8	2849 1992 4841	0.9 0.5 0.7	3.3 2.5 2.9
Eastern Asia	Male Female Total	60979 46392 107371	2.5 2.7 2.6	5.5 3.6 4.5	58037 45080 103117	3.3 4.5 3.7	5.2 3.4 4.3	42421 30779 73200	1.0 0.8 0.9	6.4 4.8 5.6
Australia/ New Zealand	Male Female Total	1778 1572 3350	2.2 2.5 2.3	7.5 5.4 6.5	1520 1493 3013	5.2 6.5 5.8	6.3 5.0 5.6	950 825 1775	0.4 0.4 0.4	8.7 7.4 8.0

* Age-standardized rate (W): A rate is the number of new cases or deaths per 100 000 persons per year. An age-standardized rate is the rate that a population would have if it had a standard age structure. Standardization is necessary when comparing several populations that differ with respect to age because age has a powerful influence on the risk of cancer.

**Figure 2:**
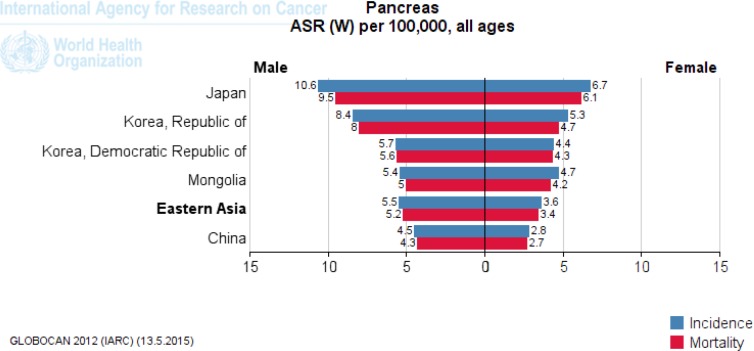
The incidence and mortality of pancreatic cancer in Eastern Asia, according to GLOBOCAN estimation project 2012

**Figure 3.**
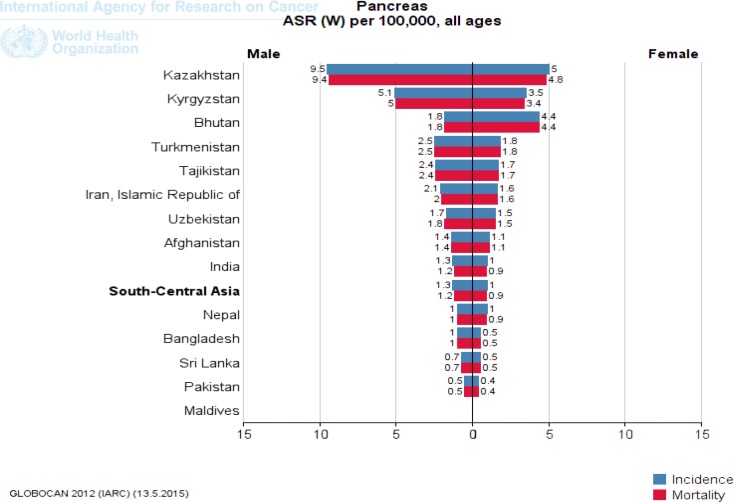
The incidence and mortality of pancreatic cancer in Central Asia, according to GLOBOCAN estimation project 2012

**Figure 4.**
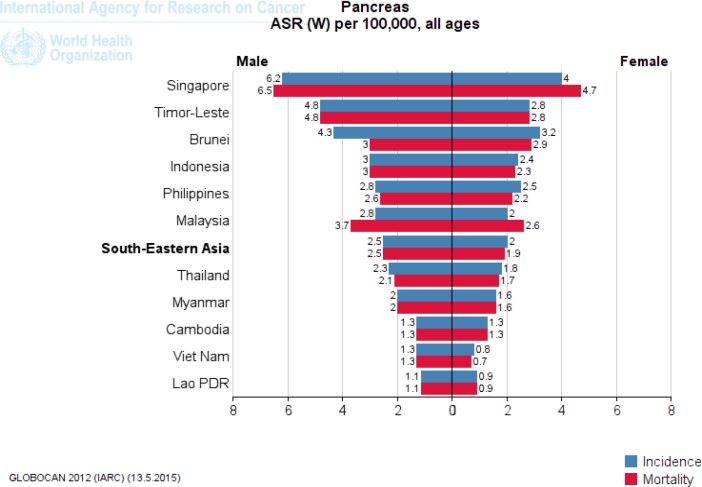
The incidence and mortality of pancreatic cancer in Southeast Asia, according to GLOBOCAN estimation project 2012

**Figure 5:**
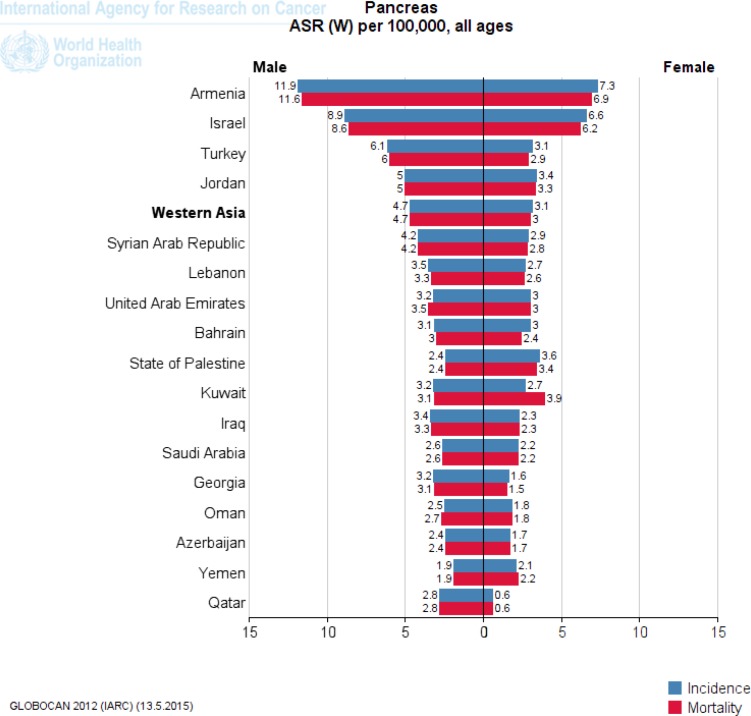
The incidence and mortality of pancreatic cancer in Western Asia, according to GLOBOCAN estimation project 2012

Japan in Eastern Asia (8.5), Kazakhstan in Central Asia (6.8), Singapore in Southeast Asia (5.1), Armenia in Western Asia (9.3) and Australia in Oceania (6.6) have a high ASR incidence of pancreatic cancer per 100,000 (Figure2-5). Figure 2 to 5 provide the incidence and mortality of pancreatic cancer in different regions of Asia for male and female. Armenia and Japan have the highest mortality rate due to pancreatic cancer in Asia-Pacific region (Table 2). In addition, the World Bank ranking was showing in table 2 for countries in Asia and the Pacific in terms of mortality among the countries of the world. Iran among Asian countries in rms of incidence and mortality of pancreatic cancer is ranked eleventh (5). Pancreatic cancer is the twelfth leading cancer to death in Iran (5). Trend of pancreatic cancer mortality in Iran slightly decreased in recent decade (11). Nonetheless, it is impossible to fully explain the differences in the incidence of pancreatic cancer in different parts of the world; most of the international variation in the incidence of pancreatic cancer has been attributed to exposure to known or suspected risk factors related to lifestyle or the environment.

**Table 2:** Highest mortality rate in twenty countries in Asia-Pacific region and status of Iran

countries	Regions	Mortality rate per 100,000	World Rank
Armenia	Western Asia	13.88	1
Japan	East Asia	9.35	13
Kazakhstan	Central Asia	7.50	38
South Korea	East Asia	7.39	39
Australia	Oceania	6.77	46
New Zealand	Oceania	6.17	49
Singapore	Southeast Asia	5.92	52
North Korea	East Asia	5.65	54
China	East Asia	4.13	70
Arab Emirates	Western Asia	4.12	71
Jordan	Western Asia	4.08	74
Kyrgyzstan	Central Asia	3.95	78
Syria	Western Asia	3.61	83
Malaysia	Southeast Asia	3.54	85
Indonesia	Southeast Asia	3.22	90
Bahrain	Western Asia	2.77	95
Iraq	Western Asia	2.75	96
Kuwait	Western Asia	2.62	100
Azerbaijan	Western Asia	2.46	105
Iran	Western Asia	1.95	119

Data From: World Health Ranking 2014; http://www.worldlifeexpectancy.com/asia/pancreas-cancer-cause-of-death

The incidence and mortality rate of pancreatic cancer in both gender has increased with age and almost 90% of all cases are diagnosed or all deaths are registered after age of 55 years (4). Pancreatic cancer still occurs in elderly people in Asia-Pacific region but compared to patients in western countries patients are relatively younger at the time of diagnosis. But similar to western countries, pancreatic cancer in this region occurs more in men than in women. This probably related to the higher prevalence of smoking and some other risk factors among males (12, 13). The results of study by Ansary Moghadam et al. in Asia-Pacific region showed that smoking, obesity and diabetes were important and they are potentially modifiable risk factors for pancreatic cancer in this region. The age-adjusted hazard ratios (HR) for mortality of pancreatic cancer; due to smoking and diabetes were (HR: 1.61; 95% CI, 1.12-2.32) and (HR: 1.76; 95%CI, 1.15-2.69) respectively (P<0.05); these results were similar for men and women. But in men, combination of smoking and diabetes was more effective on pancreatic cancer (HR: 2.47; 95%CI, 1.17-5.21) (14).

**Survival of pancreatic cancer**

The survival rate of pancreatic cancer is short; the relative one ear survival rate is only 24%, and the overall 5-year survival is about 6% (ranges from 2% to 9%) (15). Survival rates of pancreatic cancer in the community are affected by many factors, such as age, sex, type of cancer, staging at the time of diagnosis, serum albumin level, and tumor size. Age and tumor characteristics can have effect on survival of pancreatic cancer (16, 17); advancing of the age and advancing of the grade of abdominal lymph node and liver metastasis were all associated with poorer survival (18, 19). Moreover, the type of pancreatic cancer can also have effect on survival, according to the nine population-based cancer registries from 1973-2000; endocrine pancreatic cancer survival is longer than in patients with exocrine tumors with a 0.28-fold lower risk of mortality (95% CI, 0.26-0.30). These results were similar for both sexes (20).

**Risk factors of pancreatic cancer**

Several personal and environmental factors have been associated with pancreatic cancer. They are divided into two categories: modifiable and non-modifiable risk factors. Modifiable risk factors include smoking, obesity, alcohol and dietary factors. Risk factors that are not modifiable include gender, age, family history of pancreatic cancer, genetic factors, diabetes mellitus, chronic pancreatitis and chronic infections (21). Most host factors are non-modifiable whereas the environmental factors are modifiable risk factors. Recently systematic review on results of 117 meta-analytical or pooled data reported that smoking as strong evidence and helicobacter pylori infection as moderate evidence, with respective population attributable fractions of 11-32% and 4-25% were the major risk factors for pancreatic cancer worldwide, while the major protective factors were history of allergy and increasing fruits and vegetables intake with respective population preventable fraction of 3-7% and 0-12% (22).

**Modifiable risk factors**

- Smoking: Smoking is the most important environmental factor for pancreatic cancer in the world, approximately 25% to 35% of pancreatic cancers are associated with Cigarette smoking. The risk of pancreatic cancer is nearly two times higher in smokers than in non-smokers (23-25). Based on meta-analysis from the Asia-Pacific that included 30 cohort studies, current smokers had a 60% increased risk of pancreatic cancer compared to those who never had smoked (HR 1.61; 95% CI, 1.12–2.32) (14). According to previous studies, the summary relative risk (SRR) of pancreatic cancer was (1.6-2.2) for current smokers and (1.1-1.2) for former smokers (26-28). It should be noted that, the risk of pancreatic cancer will be increased in countries where smoking rates are high. Based on George Institute of Global Health in 2010; 30% of the world’s smokers was located in Asia-Pacific region. The world’s two most populous nations; India with 275 million and China with 300 million smoker users are home to more smokers than the entire population of the Europe (29). And also the rate of smoking in male of Asia and the Pacific are 8-fold higher than females (30). 

- Obesity: Obesity has associated with increased risk for plenty of different types of cancer including pancreatic cancer (31). Obesity especially central type, has been associated with a higher incidence of pancreatic cancer (32, 33). It was found that being overweight (body mass index ≥25 kg/m2) or obese (BMI≥30 kg/m2) during early adulthood may be associated with a greater risk of pancreatic cancer (34). In addition, a previous meta-analysis in Asia-Pacific region evaluated the positive association with central obesity (2-cm higher waist circumference) with an 8% (95% CI, 2-15%) greater risk of pancreatic cancer (14). Obese individuals have a 20% higher risk of developing pancreatic cancer than those who have normal range weight (35). Unfortunately, overweight and obesity are endemic in most of the Asia-Pacific region (36). 

- Alcohol: Based on many previous studies, the risk of pancreatic cancer is undoubtedly increased by high alcohol consumption (more than 3 drinks per day), whereas they did not find any association between the risk of pancreatic cancer and low-to-moderate alcohol intake (37-39). The status of alcohol consumption in Asia-pacific region is completely different; in developed countries of this region such as Australia, New Zealand, South Korea, Japan, and china the rate of alcohol consumption is equal to European countries over 7 liters of alcohol per capita in 2009 (40) while, the other part of this region especially in Middle-East and Western of Asia where the Moslem countries located because of cultural and religious traditions drinking of alcohol is forbidden, so the consumption in these areas is minimal. Therefore, we could not find any association between alcohol and the risk of pancreatic cancer in these areas of Asia. 

- Alcohol and Smoking: The association between alcohol and smoking is very close, so it is really difficult to implicate alcohol as an independent risk factor for pancreatic cancer. Based on recent study; heavy alcohol consumption was associated with a significantly increased pancreatic cancer risk (age-adjusted OR=4.04, 95% CI: 1.58, 10.37), whereas this significant association with heavy drinking was not observed among non-smokers (age-adjusted OR=2.01, 95% CI: 0.50, 8.18). Furthermore, low-to-moderate alcohol intake was associated with increased pancreas cancer risk among current smokers (41).

- Dietary factors: It seems reasonable that diet would affect the risk of different digestive diseases and cancers, including those of the pancreas that the dietary factors up to 30–50% impact on pancreatic cancer (21, 42). There are some evidence that the consumption of red meats (particularly when cooked at high temperatures), cholesterol, fried foods and other foods containing nitrosamines increase the risk of pancreatic cancer (43, 44).

A positive association between consumption of red meats and processed meats with risk of pancreatic cancer is biologically acceptable. Carcinogens in met and nitrite or N-nitroso compounds that used for preserved processed meats that can reach the pancreas via the bloodstream may be involved in pancreatic cancer (45). The results of meta-analysis that included 11 case-control studies showed the red meat consumption increased the pancreatic cancer risk by 48% (95% CI=1.25-1.76). On the other hand, high intake of vegetables and fruits especially those enrich in citrus and antioxidants are inversely associated with risk of pancreatic cancer, decreasing the risk by 38% (95% CI=0.54-0.73) and 29% (95% CI=0.59-0.84), respectively (46). In addition, another meta-analysis of 11 prospective studies, with 6643 pancreatic cancer cases, found a positive association between pancreatic cancer incidence and processed meat consumption. High consumption of red meat (120 g/day) was associated with increased risk of PC with the risk of PC (RR=1.13, 95% CI: 0.93–1.39) and also increased consumption of processed meat (50 g/day) was responsible for a 20% increased risk of PC (RR=1.19, 95% CI: 1.04–1.36) (47). But some studies have not supported these findings (48) or have provided support for the association among men only (49).

**Non- Modifiable risk factors**

- Gender: pancreas cancer is more common in males than in females (50). Globally, the age-standardized rate (ASR) incidence for pancreatic cancer is 4.9/100,000 for men and 3.6/100,000 for women (8). The incidence rates also differ among men and women in Asia; 3.8/100,000 in men and 2.6/100,000 in women are observed in Asia region (8). Pancreatic cancer occurs more in men possibly due to environmental or occupational risk factors as well as lifestyle such as heavy smoking habit and alcohol intake in men, although it is possible that there may yet be undiscovered genetic factors influencing cancer incidence and mortality in males and females.

- Age: The risk of developing pancreatic cancer is increased by age, more than 80% of cases are diagnosed at 60 and 80 years old (4). With only <10% of cases occurring in individuals under 50 years of age and is rare in people under 25 years old (16). The majority of pancreatic cancer patients in Asia and Oceania were 65 years or older, where 62% of Asia patients and 70% of Oceania patients were 65 years old or more (5). This was in accordance with the results reported in SEER Cancer Statistics Review that pancreatic cancer is predominately a disease of older individuals and almost all patients are older than fifty (51).

- Ethnicity: the incidence of pancreatic cancer is different in racial disparities. Many studies have been reported the significant differences in the incidence of pancreatic cancer between races (52-54). Pancreatic cancer incidence rates for African-Americans are higher than Caucasians, while the incidence is lowest in Asian-Americans and Pacific Islanders (55). Generally, the risk of pancreatic cancer rate is considerably higher in Blacks than in any other racial group (50). Differences in incidence of pancreatic cancer between races can be attributed to modifiable risk factors such as diet, alcohol, smoking, and vitamin D insufficiency. Nevertheless, some population based studies have reported that racial disparities in pancreatic cancer is not completely explained by the known and suspected risk factors. In addition other factors such as genetic factors, acquired mutations from known toxins e.g. the ability to detoxify tobacco products, oncogene mutation and biomarker immune expression may contribute to the increased risk of pancreatic cancer (56, 57). In studies comparing the oncogene mutations and biomarker immune expression among Chines, Japanese and Western patients, results have shown that a gene component to pancreatic cancer might be different between Asian and Western pancreatic cancer; Asian patients with pancreatic cancer have different expressions of KRAS and p53 than Western patients (58, 59). These findings suggest that these differences in genetic and molecular related to race can affect the incidence of pancreatic cancer, and may also explain the difference in survival rates after treatment of pancreatic cancer in racial disparities. In general, it seems that Asian patients have a better survival rate than non-Asian patients (60). 

- Family history: Family history is one of the pancreatic cancer risk factor (61). About 5% to 10% of individuals with pancreatic cancer report a history of pancreatic cancer in first-degree relatives (62). In meta-analysis of seven case-control and two cohort studies showed a significant increase in pancreatic cancer risk associated with having an affected relative with summery relative risk (RR= 1.80, 95% CI, 1.48-2.12) (61). Also in nested case-control study of pooled data from 10 cohort studies reported that a family history of pancreatic cancer in a close family member was associated with increased risk of pancreatic cancer (OR=1.76, 95% CI, 1.19-2.61) (63).

- Genetic factors: Genetic variation or mutation (Germ-line mutation) plays an important role in increased risk of pancreatic cancer (64). Approximately, 10% of patients with pancreatic cancer have some genetic predisposition such as gene variations or alterations to developing the disease (65). Several germ-line mutations have been identified to be involved in hereditary forms of PC, including both familial PC (FPC) and PC as one of the manifestations of a hereditary cancer syndrome or other hereditary conditions (BRCA1, BRCA2, PALB2, ATM, CDKN2A, APC, MLH1, MSH2, MSH6, PMS2, PRSS1 and STK11) (66). Pancreatic cancer is also found to be associated with a number of familial cancer syndromes such as Lynch syndrome, Peutz-Jeghers syndrome, the Familial atypical multiple mole melanoma syndrome, Hereditary breast and ovarian cancer syndrome, Li- Fraumeni syndrome, Familial adenomatous polyposis (67). Furthermore, four main genes in inherited genetic mutations that have special role in increased risk of pancreatic cancer include; KRAS, CDKN2A (p16), p53, and SMAD4 (68). 

- Diabetic mellitus: The positive association between both type Ⅰ and Ⅱ diabetes and the risk of pancreatic cancer has been reported in numerous studies (69, 70). Diabetes mellitus may be associated with a 1.8-fold increase in the risk of developing pancreatic cancer, particularly in Hispanic men and Asians in comparison with Whites and Blacks (71, 72). Unfortunately, almost one fifth of people with diabetes globally live in just seven countries in South-East Asia and the Western Pacific; in those countries, 132 million adults have diabetes, the largest number in any region of the world (73).

- Chronic pancreatitis: The relationship between chronic pancreatitis (CP) and pancreatic cancer have been reported in previous studies (74, 75). CP is uncommon, only about 4% of these patients will develop pancreatic cancers within 20 years of diagnosis (76, 77). The prevalence of CP is very high in India (114-200/100000 population) in contrast to the Japan (4.2/100000 population) (78, 79). Chronic pancreatitis has several causes such as, hereditary and idiopathic but alcohol abuse is the commonest cause of it (80). Alcohol consumption has been increasing in the developing countries and is the most common etiological factor of CP in Australia, Japan, China and India (78, 81). 

- Infection: Few studies have been reported the association between pancreatic cancer with some chronic infections such as hepatitis B virus (HBV), hepatitis C virus (HCV) and Helicobacter pylori (82, 83). The global prevalence of HBV infection also varies widely, in Asian countries divided in three groups; low, intermediate and high endemic areas of HBV. China is now the only country in Asia that classified as high endemic of HBV. Intermediate endemic areas in Asia include; India, Taiwan, Thailand, Philippines, Korea, Iraq and United Arab Emirates, and countries with low endemicity include Japan, Pakistan, Singapore, Sri Lanka, Bangladesh, Malaysia, Iran, Kuwait and Bahrain (84). The highest prevalence rate of HCV occurs in African and Asian countries (5.3% in Africa and 2.15%-3.9% in Asia) (85). The prevalence of HCV infection in Asia-Pacific region is varies but in Japan, Saudi Arabia, Egypt and Pakistan HCV is high (84). The incidence rate of H. pylori varies by region however; it is highly prevalent in Asia and developing countries (86). A meta-analysis of 7 studies reported an increased risk of pancreatic cancer in people infected with H. pylori (87). Nevertheless, these data are not sufficient to confirm, so further studies evaluating this association are needed.

- Blood group: Numerous epidemiological studies have found an association between ABO blood groups and the risk of developing pancreatic cancer (88, 89). Based on two meta-analyses; the risk of PC about 30 to 40% will be increased among people with non-O blood group (90, 91). People with blood groups A, AB, or B have a higher risk of developing pancreatic cancer than people with blood O group; (the OR for pancreatic cancer in subjects with types A, AB, and B were (OR=1.38, 95% CI, 1.18–1.62), (OR=1.47, 95% CI, 1.07– 2.02, and OR=1.53, 95% CI, 1.21–1.92), respectively (91). 

## Conclusion

In summary, pancreatic cancer is less common than the lung, breast, stomach, liver, bowel and prostate cancer. Nonetheless, because of high mortality rate and 7th rank in the world, it remains as a challenging disease to diagnose and treat. In some countries in Asia-Pacific region, such as Armenia, Japan, Kazakhstan, New Zealand, Australia and Korea, the mortality rate of pancreatic cancer are high but, in other countries such as China the death rate due to pancreatic cancer was rising and the peak mortality might arrive in future. For the prevention of pancreatic cancer first, it is necessary to understand the epidemiology, etiology and identifying its risk factors and second, it is needed to screen and identify high-risk individuals for pancreatic cancer. 
